# Effective Detection of the 2009 H1N1 Influenza Pandemic in U.S. Veterans Affairs Medical Centers Using a National Electronic Biosurveillance System

**DOI:** 10.1371/journal.pone.0009533

**Published:** 2010-03-04

**Authors:** Patricia Schirmer, Cynthia Lucero, Gina Oda, Jessica Lopez, Mark Holodniy

**Affiliations:** 1 Office of Public Health Surveillance and Research, Department of Veterans Affairs, Palo Alto, California, United States of America; 2 Division of Infectious Diseases & Geographic Medicine, Stanford University School of Medicine, Stanford, California, United States of America; Duke University, United States of America

## Abstract

**Background:**

The 2008–09 influenza season was the time in which the Department of Veterans Affairs (VA) utilized an electronic biosurveillance system for tracking and monitoring of influenza trends. The system, known as ESSENCE or Electronic Surveillance System for the Early Notification of Community-based Epidemics, was monitored for the influenza season as well as for a rise in influenza cases at the start of the H1N1 2009 influenza pandemic. We also describe trends noted in influenza-like illness (ILI) outpatient encounter data in VA medical centers during the 2008–09 influenza season, before and after the recognition of pandemic H1N1 2009 influenza virus.

**Methodology/Principal Findings:**

We determined prevalence of ILI coded visits using VA's ESSENCE for 2008–09 seasonal influenza (Sept. 28, 2008–April 25, 2009 corresponding to CDC 2008–2009 flu season weeks 40–16) and the early period of pandemic H1N1 2009 (April 26, 2009–July 31, 2009 corresponding to CDC 2008–2009 flu season weeks 17–30). Differences in diagnostic ICD-9-CM code frequencies were analyzed using Chi-square and odds ratios. There were 649,574 ILI encounters captured representing 633,893 patients. The prevalence of VA ILI visits mirrored the CDC's Outpatient ILI Surveillance Network (ILINet) data with peaks in late December, early February, and late April/early May, mirroring the ILINet data; however, the peaks seen in the VA were smaller. Of 31 ILI codes, 6 decreased and 11 increased significantly during the early period of pandemic H1N1 2009. The ILI codes that significantly increased were more likely to be symptom codes. Although influenza with respiratory manifestation (487.1) was the most common code used among 150 confirmed pandemic H1N1 2009 cases, overall it significantly decreased since the start of the pandemic.

**Conclusions/Significance:**

VA ESSENCE effectively detected and tracked changing ILI trends during pandemic H1N1 2009 and represents an important temporal alerting system for monitoring health events in VA facilities.

## Introduction

As a result of genetic reassortment, a novel influenza A virus emerged in 2009 as the source of a global influenza pandemic, as declared by the World Health Organization (WHO) on June 11, 2009[1], [2], [3]. On July 24, 2009, CDC discontinued official reporting of individual cases of confirmed and probable pandemic H1N1 2009 infection[4]. As of July 31, 2009, the Center for Disease Control and Prevention (CDC) had reported 5,514 hospitalized cases and 353 deaths from pandemic H1N1 2009 influenza in over 40 U.S. States and territories, while worldwide, more than 160 countries reported over 160,000 confirmed cases and over 1,100 deaths[5], [6].

The concern for a global influenza pandemic resulted in public health officials worldwide reemphasizing the need to use biosurveillance systems for monitoring such events. Biosurveillance systems monitor available data sources for outbreaks of a disease before identifying symptoms are confirmed [7]. Currently, there are several biosurveillance systems that exist for monitoring potential public health concerns such as pandemic influenza. The CDC uses a combination of sentinel providers manually reporting encounters with ill patients to the US Outpatient Influenza-like Illness Surveillance network (ILINet), which is the only national surveillance of influenza-like illness that is available publically, and a web-based system, BioSense, which is used by healthcare facilities as well as state and local public health officials to detect possible outbreaks[8], [9]; whereas the Department of Veterans Affairs (VA), Department of Defense (DoD) and many state and local public health departments use the Electronic Surveillance System for the Early Notification of Community-based Epidemics (ESSENCE) which uses algorithms to detect potential outbreaks based on either *International Classification of Diseases, Clinical Modification, 9^th^ Revision* (ICD-9-CM) codes or chief complaint data[10]. Other surveillance systems include the New York City surveillance system, Australian Sentinel Practice Research Network (ASPREN) as well as computer rule-based systems (Expert System Platform®, Theradoc) that rely on patient information extracted from an electronic medical record (EMR) [11], [12], [13], [14]. More novel approaches such as monitoring influenza internet searches using Google Flu or medical telephone call center data also provide important predictive information of emerging influenza trends [2], [15], [16], [17], [18], [19], [20].

While many systems have been developed for biosurveillance, there is minimal data published on the validity of or comparisons between such systems. In addition, systems use varied definitions for influenza or influenza-like illness (ILI), and depending on the source of information, symptoms, physical findings or laboratory tests included could underestimate or more accurately predict those with confirmed influenza[21], [22], [23], [24], [25], [26]. Although other approaches such as natural language processing free-text extraction or utilization of over-the-counter pharmaceutical sales, number of emergency room visits, absenteeism and triage telephone calls appear to improve the timeliness of detection, no strong conclusions could be made as to the best data indicator[27], [28]. The 2008–09 influenza season was the first flu season in which VA utilized the ESSENCE system for weekly influenza monitoring and surveillance. Herein, we describe the successful performance of VA ESSENCE for detection of 2008–09 seasonal and pandemic H1N1 2009 influenza in VA healthcare facilities. We further describe the frequencies and differences in ICD-9-CM coding during these two periods, and reviewed the ICD-9-CM codes of VA patients identified with laboratory-confirmed pandemic H1N1 2009 influenza infection.

## Methods

VA ESSENCE extracts ICD-9-CM diagnosis codes and demographic data from all outpatient and emergency department (ED) visits from all 153 VA hospitals and 774 community-based outpatient clinics (CBOCs) in all 50 states, Puerto Rico, U.S. Virgin Islands, Guam, American Samoa, and the Philippines. A limited number of inpatient visits and consultations are also captured. ICD-9-CM codes can be analyzed individually or grouped into syndrome categories (e.g. ILI, gastrointestinal, or hemorrhagic illness). Analysis is performed through complex algorithms utilizing spatial and temporal data-aggregation strategies to detect when observed counts are significantly above predicted values [7]. Analyses may be performed for a single facility on a single day, but can also be performed for regions, multiple facilities (e.g. entire Veteran Integrated Service Network, VISN) or nationally for the VA over various time periods (e.g. week, month, year etc.), as designated by the system user.

VA ESSENCE determines an expected count by employing regression modeling based on historical data, day-of-the week effects, seasonal trends and effects due to other factors[7]. After determining the expected count, the system applies statistics and runs significance tests for each syndrome or ICD-9-CM code to determine whether the observed counts are reasonably close to what is expected from model predictions[7]. When the test for reasonable agreement fails, VA ESSENCE produces alerts to indicate a count that is significantly above the predicted level. Alerts are designed to warn the system user of a possible outbreak or cluster. Tests for reasonable agreement employ confidence intervals (CIs) and when the observed count falls between the 95% and 99% CI a low-level (yellow) alert is generated. If the count exceeds the 99% CI, a high-level (red) alert is triggered.

Thirty-one codes constitute the ILI syndrome group, which is further broken down into the following 9 subgroups - bronchitis, cough, disease of the upper respiratory tract, fever, influenza, pneumonia, sore throat, URI, and viral infection (Table 1 and 2). Visits were counted as ILI if they had at least one ICD-9-CM diagnostic code included in the 31 codes that define the ILI syndrome group but these do not necessarily represent laboratory-confirmed influenza cases. A complete list of ICD-9-CM codes for the ILI syndrome group is presented in Table 1.

**Table 1:** ICD-9-CM Codes for Influenza-like Illness (ILI) Diagnosis, comparing seasonal versus early period of pandemic H1N1 2009.

ICD-9-CM Code Description	ICD-9-CM Code	Total for 9/28/08-4/25/09	Percent Total/Individual	Percent Total/ILI	Total for 4/26/09-7/31/09	Percent Total/Individual	Crude OR	CI	P Value
Viral Infection NEC	079.89	202	0.04	0.04	130	0.08	1.80	1.44–2.26	**1.00E-07**
Viral Infection NOS	079.99	5031	1.05	1.07	2467	1.44	1.38	1.31–1.45	**<1.00E-07**
Nasopharyngitis, acute	460	12490	2.61	2.66	2606	1.52	0.58	0.55–0.60	**<1.00E-07**
Pharyngitis, acute	462	35719	7.46	7.62	14758	8.63	1.17	1.15–1.19	**<1.00E-07**
Laryngitis, acute, without obstruction	464.00	1426	0.3	0.30	565	0.33	1.11	1–1.22	**0.03769**
Tracheitis, acute, without obstruction	464.10	221	0.05	0.05	74	0.04	0.94	0.71–1.23	0.6273
Laryngotracheitis, acute without obstruction	464.20	20	0.004	0.004	8	0.005	1.12	0.45–2.67	0.7877
Laryngopharyngitis, acute	465.0	113	0.02	0.02	48	0.03	1.19	0.84–1.69	0.31543
Infectious upper respiratory, multiple sites, acute NEC	465.8	186	0.04	0.04	71	0.04	1.07	0.80–1.42	0.63681
Infectious upper respiratory, multiple sites, acute NOS	465.9	116520	**24.35**	24.85	34244	**20.02**	0.78	0.77–0.79	**<1.00E-07**
Bronchitis, acute	466.0	66201	**13.83**	14.12	20716	12.11	0.86	0.84–0.87	**<1.00E-07**
Bronchiolitis due to RSV	466.11	10	0.002	0.002	7	0.004	1.96	0.67–5.57	0.1646
Bronchiolitis, acute, due to other infectious organism	466.19	278	0.058	0.059	94	0.055	0.95	0.74–1.20	0.642
Disease, upper respiratory NEC/NOS	478.9	431	0.09	0.09	169	0.099	1.1	0.91–1.32	0.307
Pneumonia due to adenovirus	480.0	0	0	0.00	1	0.0006	NA	NA	0.943
Pneumonia due to RSV	480.1	1	0.0002	0.0002	2	0.001	5.6	0.4–155.7	0.113
Pneumonia due to parainfluenza	480.2	3	0.0006	0.0006	0	0.00	0	0–6.23	0.3
Pneumonia due to virus NEC	480.8	23	0.005	0.005	7	0.004	0.85	0.33–2.08	0.709
Viral pneumonia unspecified	480.9	76	0.016	0.016	29	0.017	1.07	0.68–1.67	0.764
Pneumonia in other infectious disease NEC	484.8	25	0.005	0.005	14	0.008	1.57	0.77–3.14	0.175
Bronchopneumonia organism NOS	485	461	0.096	0.098	127	0.074	0.77	0.63–0.94	**0.0092**
Pneumonia, organism NOS	486	58286	12.18	12.42	22121	**12.94**	1.07	1.05–1.09	**<1.00E-07**
Influenza with pneumonia	487.0	63	0.013	0.013	38	0.022	1.69	1.11–2.57	**0.00995**
Influenza with respiratory manifestation NEC	487.1	7175	1.50	1.53	1762	1.03	0.68	0.65–0.72	**<1.00E-07**
Influenza with other manifestation NEC	487.8	37	0.0077	0.0079	45	0.026	3.4	2.16–5.37	**<1.00E-07**
Bronchitis NOS	490	58007	12.12	12.37	17584	10.28	0.83	0.82–0.85	**<1.00E-07**
Fever and other physiologic disturbances of temperature regulation	780.60/780.6	15268	3.19	3.26	9213	5.39	1.73	1.68–1.77	**<1.00E-07**
Chills (without fever)	780.64	1013	0.21	0.22	483	0.28	1.34	1.20–1.49	**2.00E-07**
Hypothermia not assoc with low environmental temperature	780.65	103	0.022	0.022	29	0.017	0.79	0.51–1.21	0.255
Pain, throat	784.1	2949	0.62	0.63	1523	0.89	1.45	1.36–1.54	**<1.00E-07**
Cough	786.2	96206	**20.1**	20.51	42095	**24.61**	1.3	1.28–1.31	**<1.00E-07**

**Table 2:** ICD-9-CM Codes for Influenza-like Illness (ILI) Subgroups, comparing seasonal versus early period of pandemic H1N1 2009.

ICD-9-CM Grouping Description	ICD-9-CM Code	Total for 9/28/08-4/25/09	Percent Total/Individual	Percent Total/ILI	Total for 4/26/09-7/31/09	Percent Total/Individual	Crude OR	CI	P Value
Bronchitis	466.0, 466.11, 466.19, 490.	124496	**26.02**	26.55	38401	**22.45**	0.82	0.81–0.83	**<1.00E-07**
Cough	786.2	96206	**20.10**	20.51	42095	**24.61**	1.3	1.28–1.31	**<1.00E-07**
Disease of Upper Respiratory Tract	478.9	431	0.09	0.092	169	0.099	1.1	0.91–1.32	0.3066805
Fever	780.6, 780.60, 780.64, 780.65	16384	3.42	3.49	9725	5.686	1.17	1.66–1.75	**<1.00E-07**
Influenza	487.0, 487.1, 487.8	7275	1.52	1.55	1845	1.08	0.71	0.67–0.74	**<1.00E-07**
Pneumonia	480.0, 480.1, 480.2, 480.8, 480.9, 484.8, 485, 486	58875	12.30	12.55	22301	13.04	1.07	1.05–1.09	**<1.00E-07**
Sore Throat	784.1	2949	0.62	0.63	1523	0.89	1.45	1.36–1.54	**<1.00E-07**
URI	460., 462., 464.00, 464.10, 464.20, 465.0, 465.8, 465.9	166695	**34.83**	35.55	52374	**30.62**	0.83	0.82–0.84	**<1.00E-07**
Viral Infection	079.89, 079.99	5233	1.09	1.11	2597	1.52	1.39	1.33–1.46	**<1.00E-07**

The percentage of visits for ILI was calculated weekly and compared to CDC's ILINet, which is not a gold standard but is currently the only available national influenza-like illness surveillance for the US. ILINet consists of over 3,000 voluntary providers in all 50 states, the District of Columbia, and the US Virgin Islands reports approximately 25 million patient visits each year[9]. Each week, approximately 1,400 outpatient sites from around the country manually report the total number of patients seen and the number by age group with ILI symptoms. ILINet does not collect actual patient age or gender. For the CDC system, ILI is defined as fever (temperature of 100°F [37.8°C] or greater) and a cough and/or sore throat in the absence of a known cause other than influenza[9], [29]. ILINet sites with electronic health records use an equivalent definition as determined by the state public health authorities[9]. The percentage of patient visits to these healthcare providers for ILI reported each week are weighted on the basis of state population and is compared with the national baseline of 2.3%[9]. The national baseline is the mean percentage of patient visits for ILI during non-influenza weeks for the previous 3 seasons plus 2 standard deviations [9].

We divided our observations into 2 periods: the 2008–09 seasonal influenza period (Sept. 28, 2008 to April 25, 2009 corresponding to CDC 2008–2009 flu season weeks 40–16) and the early pandemic H1N1 2009 period (April 26, 2009 to July 31, 2009 corresponding to CDC 2008–2009 flu season weeks 17–30) since April 26^th^ was the date of the first confirmed pandemic H1N1 2009 case within the VA system.

VA ESSENCE was queried for the ILI syndrome group as well as the individual ICD-9-CM codes that comprise the ILI group for both time periods. Data was downloaded and compiled while graphs were downloaded directly from the VA ESSENCE system for the above dates. We reviewed charts from the first 150 patients identified from 44 VA hospitals with laboratory-confirmed pandemic H1N1 2009 influenza and evaluated for ICD-9-CM codes and for whether they were detected with VA ESSENCE. Descriptive statistics where used to calculate frequencies of specific ICD-9-CM codes. The CDC's Epi Info, was used to calculate Mantel-Haenszel Chi Square testing for proportional analyses comparing frequencies of ICD-9-CM codes and syndrome subgroups during seasonal and pandemic H1N1 2009 outbreak periods and to generate odds ratios (OR) and confidence intervals (CI).

This project was approved by the Stanford University Institutional Review Board. The Human Subjects Research Panel at Stanford University determined that the study entitled “Healthcare-Associated Infections and Syndromic Surveillance in the Department of Veterans” met the requirements of regulation OHRP 45 CFR 46.116 (d): Requests for waiver or alteration of the informed consent process, in research that is not subject to FDA regulation in that: (1) The research involved no more than minimal risk to the subjects; (2) the waiver or alteration would not adversely affect the rights and welfare of the subjects; (3) the research could not practicably be carried out without the waiver or alteration; and (4) the subjects would be provided with additional pertinent information after participation. This study was approved because the data used for its conduct was retrospective and would be obtained through subject EMR from primary care doctors, thus, it was not anticipated that any situation would arise in which pertinent information would need to be shared with individual subjects. We publish findings from this study and established the database regarding infection control as a tool for providers, thus patients would learn and benefit from this study through the care of their primary care doctors.

## Results

**Figure 1:**
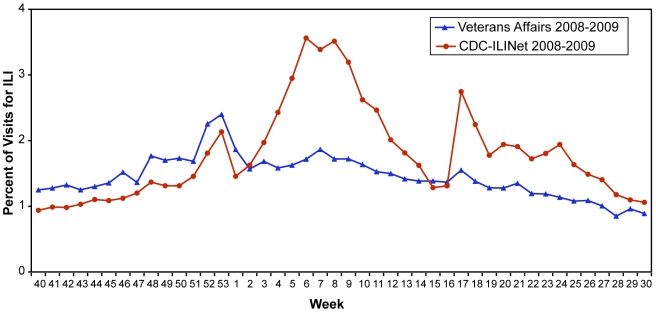
VA ILI cases compared to CDC ILINet from Sept. 28, 2008-July 31, 2009 (corresponding to CDC 2008–2009 flu season weeks 40–30). Observations are divided into 2 periods: the 2008–09 seasonal influenza period (Sept. 28, 2008 to April 25, 2009 corresponding to CDC 2008–2009 flu season weeks 40–16) and the early pandemic H1N1 2009 period (April 26, 2009 to July 31, 2009 corresponding to CDC 2008–2009 flu season weeks 17–30) since April 26^th^ was the date of the first confirmed pandemic H1N1 2009 case within the VA system.

Currently, there are 7.84 million veterans enrolled and 5.58 million who received healthcare in the VA system in 2008 at 153 VA medical facilities plus community-based outpatient clinics (CBOCs) in all 50 states, Puerto Rico, U.S. Virgin Islands, Guam, American Samoa, and the Philippines[30]. For the 2008–09 influenza season extending to week 30 (ending August 1, 2009), 633,893 unique veteran patients had approximately 694,574 visits with an ILI diagnoses reported in VA ESSENCE. A comparison of the percentage of VA visits for ILI each week was calculated and compared to the CDC's ILINet (Figure 1). Visits in the VA occurred in a similar pattern to ILINet with peaks in late December and early February. Initially, the VA had a higher percentage of visits for ILI than ILINet providers, however, from week 3 onward the overall percentage of visits for ILI in the VA were lower than reported by the ILINet. Small peaks in February and late April/early May were present, mirroring the ILINet data; however, the peaks observed in VA were flatter and smaller.

**Figure 2:**
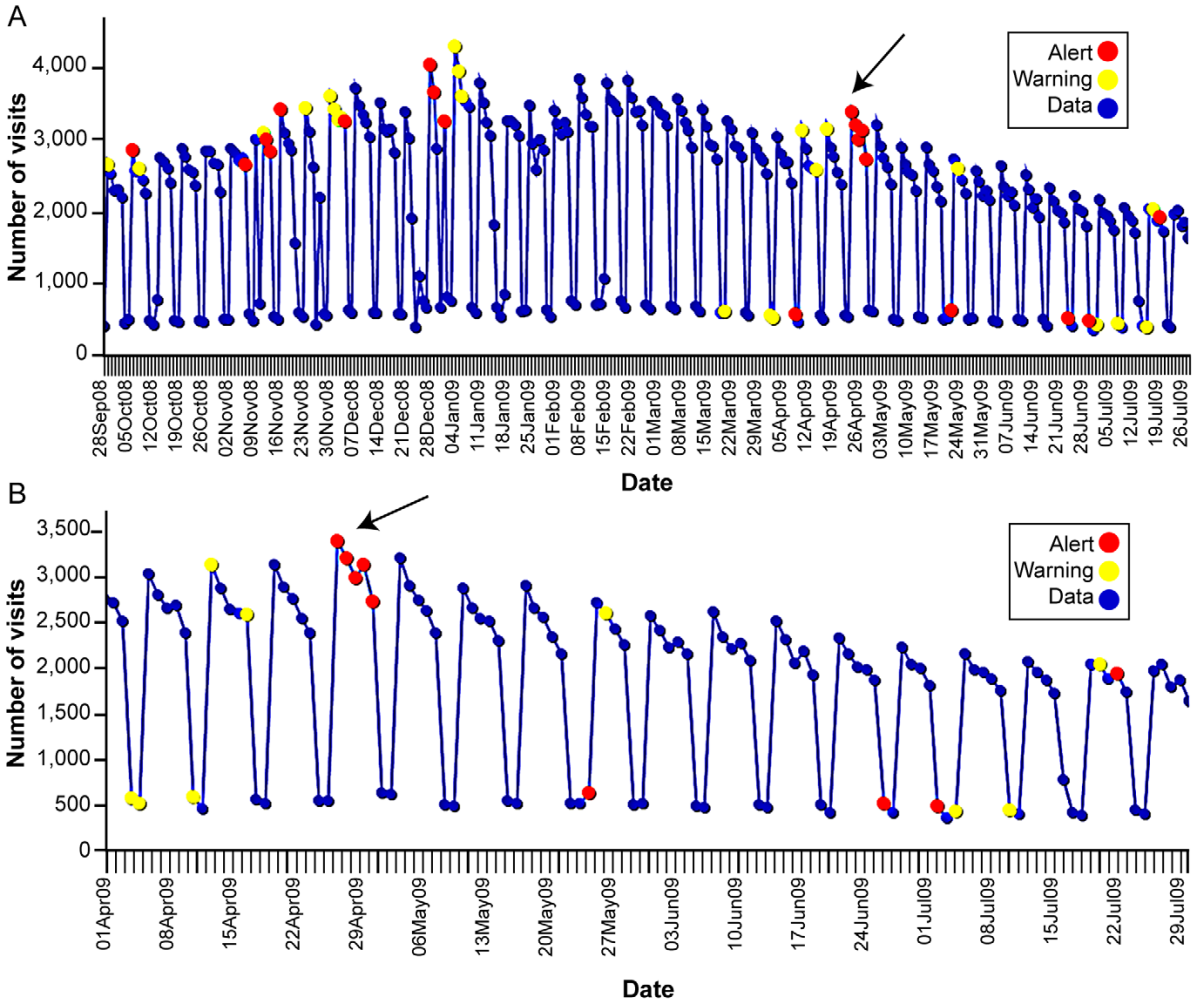
VA Outpatient Visits for ILI from the ESSENCE Surveillance System, Sept 28, 2008-July 31, 2009 (corresponding to CDC 2008–2009 flu season weeks 40–30). Data was compiled from VA's Electronic Surveillance System for the Early Notification of Community-Based Epidemics (ESSENCE). Diagnosis codes from outpatient and emergency department visits are analyzed for total number of patients with influenza-like Illness (ILI). Visits are counted as ILI if their diagnostic code is fever, an included respiratory code, or unspecified viral illness. A complete list of ICD-9-CM codes for the ILI category can be found in Table 1. *Counts do not represent confirmed influenza cases*. (A) VA outpatient visits for ILI from the ESSENCE from Sept 28, 2008-July 31, 2009 (corresponding to CDC 2008–2009 flu season weeks 40–30). (B) VA outpatient visits for ILI from ESSENCE, April 1, 2009-July 31, 2009 (corresponding to CDC 2008–2009 flu season weeks 13–30). Arrow points to the start of pandemic H1N1 2009.

**Figure 3:**
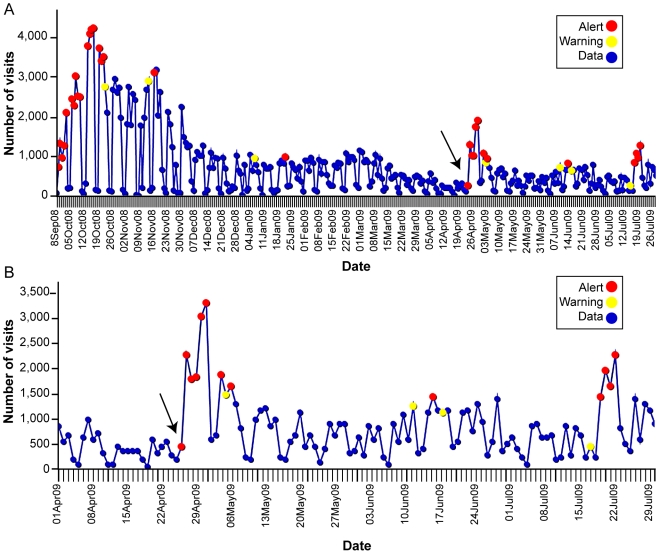
VA Outpatient Visits with Influenza (ICD-9-CM 487) from ESSENCE, Sept. 28, 2008-July 31, 2009 (corresponding to CDC 2008–2009 flu season weeks 40–30). Data was compiled from VA's Electronic Surveillance System for the Early Notification of Community-Based Epidemics (ESSENCE). Diagnosis codes from outpatient and emergency department visits are analyzed for total number of patients with an influenza-specific ICD-9-CM diagnosis code (487). *Counts do not represent confirmed influenza cases*. (A) VA outpatient visits with influenza (ICD-9-CM 487) from the ESSENCE from Sept 28, 2008-July 31, 2009 (corresponding to CDC 2008-2009 flu season weeks 40–30). (B) VA outpatient visits with influenza (ICD-9-CM 487) from ESSENCE, April 1, 2009-July 31, 2009 (corresponding to CDC 2008–2009 flu season weeks 13–30). Arrow points to the start of pandemic H1N1 2009.

Visits for ILI from all VA facilities nationwide detected using VA ESSENCE from Sept. 28, 2008 to July 31, 2009 are shown in Figure 2A with the early period of pandemic H1N1 2009 displayed in Figure 2B. The saw-tooth pattern seen as regular drops in counts are due to weekend variation since the majority of VA clinics are closed on weekends and visits recorded on weekends are primarily from ED visits. The ESSENCE detecting algorithms are designed to account for this weekend variation. A similar depiction of all VA outpatient visits coded with an ICD-9-CM code specific for influenza (487), but not necessarily confirmed influenza-positive cases, from Sept. 28, 2008 to July 31, 2009 is seen in Figure 3A with the early period of pandemic H1N1 2009 demonstrated in Figure 3B. Red alerts (indicating a significant elevation in the number of influenza-coded visits in the system) were seen at the beginning of the influenza season as well as during the emergence of pandemic H1N1 2009 in late April/early May.

The frequency of each ICD-9-CM code was extracted from VA ESSENCE (Table 1). The top three ILI ICD-9-CM codes during the 2008–09 seasonal influenza period (Sept. 28, 2008-April 25, 2009 corresponding to CDC 2008–2009 flu season weeks 40–16) were acute upper respiratory infection (URI) NOS (465.9, 24%), cough (786.2, 20%), and acute bronchitis (466.0, 14%). The pandemic H1N1 2009 outbreak started in the VA on April 26, 2009 with the first confirmed case and was detected as an emergence of red alerts for the ILI syndrome group (Figure 3A and 3B). During the early pandemic H1N1 2009 period (April 26, 2009-July 31, 2009 corresponding to CDC 2008–2009 flu season weeks 17–30), the most common codes utilized were cough (786.2, 25%), acute upper respiratory infection (URI) NOS (465.9, 20%), and pneumonia, organism NOS (486, 13%). The ILI subgroups were similar for both time periods with the three most common being URI, acute bronchitis and cough (Table 2).

Frequencies for 17 of the 31 codes significantly changed after the start of pandemic H1N1 2009 at VA facilities (Table 3). Six of 17 significantly decreased during the early pandemic H1N1 2009 period while 11 significantly increased during that time (Table 3). Several of the codes which significantly increased during the early pandemic H1N1 2009 period included symptoms such as cough (786.2) [OR 1.3, p<1.00×10^−7^, CI 1.28–1.31], throat pain (784.1) [OR 1.45, p<1.00×10^−7^, CI 1.36–1.54], fever (780.6/780.60) [OR 1.71, p<1.00×10^−7^, CI 1.68–1.77], and chills (780.64) [OR 1.34, p<1.00×10^−6^, CI 1.20–1.49]. In addition, an increase in influenza with pneumonia (487.0) [OR 1.69, p<0.01, CI 1.11–2.57] and influenza with other manifestations (487.8) [OR 3.4, p<1.00×10^−7^, CI 2.16–5.37] was seen despite these remaining an overall low percentage of ILI-related visits and a decrease in the code for influenza with respiratory manifestation NEC (487.1) [OR 0.68, p<1.00×10^−7^, CI 0.65–0.72] during the early pandemic H1N1 2009 period. Interestingly, influenza with respiratory manifestation NEC (487.1) was used significantly less during the early period of the pandemic H1N1 2009 outbreak, however, it was the most common code used among the first 150 confirmed pandemic H1N1 2009 VA cases (61/150, 41%). Other ICD-9-CM codes used in confirmed cases include acute URI NOS (465.9) [39/150, 26%], fever (780.6) [19/150, 13%], pneumonia, organism NOS (486) [18/150, 12%], cough (786.2) [18/150, 12%], viral infection NOS (079.99) [9/150, 6%], acute bronchitis (466) [8/150, 5%], bronchitis NOS (490) [4/150, 3%], influenza with pneumonia (487.0) [3/150, 2%], acute pharyngitis (462) [3/150, 2%], and acute nasopharyngitis (460) [1/150, 0.7%] (Table 4). Of importance, the 150 confirmed-positive pandemic H1N1 2009 cases were not uniformly coded as influenza and only the above 11 of the 31 defined ILI codes were utilized. Of the confirmed pandemic H1N1 2009 cases, 136/150 (90.7%) were captured in the ILI syndrome group in VA ESSENCE. Seven of the 14 that were not detected in VA ESSENCE were coded with ICD-9-CM codes that were outside of the ILI syndrome group. Six of the 14 patients not captured in VA ESSENCE were employees seen in a VA occupational health clinic that are not regularly detected in the system. One of the 14 patients was directly admitted and was not seen in the outpatient or emergency department setting.

**Table 3:** ICD-9-CM codes that significantly changed since the start of pandemic H1N1 2009.

ICD-9-CM Description	ICD-9-CM Code	Change during pandemic H1N1 2009	% Before H1N1	% During H1N1	p-value	OR	CI
Acute nasopharyngitis	460	Decreased	2.61	1.52	<1.00E-07	0.58	0.55–0.60
Acute upper respiratory infection (URI) NOS	465.9	Decreased	24.35	20.02	<1.00E-07	0.78	0.77–0.79
Acute bronchitis	466.0	Decreased	13.83	12.11	<1.00E-07	0.86	0.84–0.87
Bronchopneumonia organism NOS	485	Decreased	0.096	0.074	<0.01	0.77	0.63–0.94
Influenza with respiratory manifestation NEC	487.1	Decreased	1.50	1.03	<1.00E-07	0.68	0.65–0.72
Bronchitis NOS	490	Decreased	12.12	10.28	<1.00E-07	0.83	0.82–0.85
Viral infection NEC	079.89	Increased	0.042	0.76	<1.00E-06	1.8	1.44–2.26
Viral infection NOS	079.99	Increased	1.051	1.442	<1.00E-07	1.38	1.31–1.45
Acute pharyngitis	462	Increased	7.464	8.629	<1.00E-07	1.17	1.15–1.19
Acute laryngitis without obstruction	464.00	Increased	0.298	0.330	0.038	1.11	1–1.22
Pneumonia, organism NOS	486	Increased	12.18	12.94	<1.00E-07	1.07	1.05–1.09
Influenza with pneumonia	487.0	Increased	0.013	0.022	<0.01	1.69	1.11–2.57
Influenza with other manifestation	487.8	Increased	0.0077	0.026	<1.00E-07	3.4	2.16–5.37
Fever	780.60/780.6	Increased	3.19	5.39	<1.00E-07	1.73	1.68–1.77
Chills without fever	780.64	Increased	0.212	0.282	<2.00E-07	1.34	1.20–1.49
Pain, throat	784.1	Increased	0.616	0.890	<1.00E-07	1.45	1.36–1.54
Cough	786.2	Increased	20.10	24.61	<1.00E-07	1.3	1.28–1.31

**Table 4:** ICD-9-CM Codes used for laboratory-confirmed pandemic H1N1 2009 cases in VA facilities.

ICD-9-CM Code Description	ICD-9 Code	Number
Influenza with respiratory manifestation NEC	487.1	61
Acute URI NOS	465.9	39
Fever	780.6	19
Pneumonia, organism NOS	486	18
Cough	786.2	18
Viral infection NOS	079.99	9
Acute bronchitis	466	8
Influenza with pneumonia	487.0	3
Acute pharyngitis	462	3
Bronchitis NOS	490	4
Acute nasopharyngitis	460	1

(N = 150, some cases had more than one ICD-9-CM code for the encounter).

## Discussion

VA ESSENCE effectively detected and tracked changing influenza trends within the VA nationally during seasonal influenza and the early pandemic H1N1 2009 outbreak and represents an important temporal alerting system for monitoring health events within the Veterans Health Administration (VHA). VA ILI surveillance data for the 2008–09 season followed a comparable pattern to the CDC's ILINet except that peaks in February and late April/early May were smaller than reported despite an initial increased percentage early in the influenza season. Several reasons could explain the differences seen between the VA ESSENCE ILI percentages and the CDC's ILINet percentages. First, the CDC ILINet relies on voluntary weekly reporting of ILI cases based on clinical symptoms and total patients seen by a practice while VA ESSENCE relies on daily automatic electronic extraction of ICD-9-CM codes. In addition, the ILINet solicits information from clinics that are primarily general family practice, internal medicine, pediatricians, urgent care, and emergency medicine. VA ESSENCE gathers ILI codes from all clinics across all the specialties, subspecialties as well as allied health visits including but not limited to physical therapy and occupational therapy, but no pediatric data is collected. The large numbers of clinics that are included in VA ESSENCE likely contribute to the large denominator in total patients seen which would lower the overall ILI percentage. In addition, veterans often seek healthcare more frequently than others in the community due to numerous health problems which would also contribute to the large denominator of patients seen in the VA system nationally. Unfortunately, ILINet does not collect actual patient age or gender to allow more detailed comparisons between our populations. The initial increase in ILI symptoms at the beginning of the influenza season may have been due to visits pertaining to chronic illnesses but are coded as symptoms or general diagnoses (for example cough or bronchitis) that overlap with the ILI syndrome group or there may have been another virus or organism that was contributing to the slight rise in ILI percent in the VA community. Both of these would increase the VA's ILI percentages. At this time, the comparison of ILI percent in the VA compared to the ILINet is unable to give any information regarding a difference between the seasonal influenza virus and the H1N1 2009 influenza virus, but provides a general picture of ILI percentages for the year across the nation in the VA population compared to the CDC's data.

Interestingly, during the early period of pandemic H1N1 2009 the ICD-9-CM codes applied to outpatient visits with ILI diagnosis codes shifted. While cough and acute URI remained two of the top three ICD-9-CM codes within the ILI bundle, pneumonia was slightly more frequently coded during the early pandemic period to make it the third most common ILI diagnosis during that time. Since pandemic H1N1 2009 in the general population at this time has been no more severe than seasonal influenza, patients have been urged to stay at home unless they have signs or symptoms of more severe disease such as evidence of pneumonia. Therefore, those who are seeking medical care may more frequently have pneumonia or other severe indicators of influenza. Also, there were several symptom-based codes that significantly increased during the early pandemic H1N1 2009 period including symptoms such as cough (786.2), throat pain (784.1), fever (780.6/780.60), and chills (780.64). It is unclear whether there was a reluctance on the part of the provider to diagnose a patient with influenza outside of the traditional influenza season or because they lacked accurate rapid diagnostic tests to help in determining a diagnosis. In addition, in the setting of anxiety surrounding the potential of an influenza pandemic, physicians may have more cautiously used the influenza codes.

VA ESSENCE was able to detect an increase in influenza at the start of the early pandemic H1N1 2009 period. The most frequent ICD-9-CM code during the early pandemic period was cough (786.2), while the most frequent code for patients with confirmed pandemic H1N1 2009 was influenza with respiratory manifestation NEC (487.1). However, the influenza with respiratory manifestation NEC (487.1) code was more frequently used during the period of seasonal influenza. An influenza-specific ICD-9-CM diagnosis did appear to be used for those who, based on chart review, likely had URI or influenza or documentation of rapid influenza testing. However, insufficient lab testing due to the lack of an accurate rapid diagnostic test or sparse documentation made it difficult to determine whether the patients not coded with an influenza code truly had influenza.

There were over 600,000 ILI visits in the VA system but only a small percentage of the 31 codes comprising the bundle were used. An even smaller fraction of ICD-9-CM codes in the ILI bundle were used in cases of confirmed pandemic H1N1 2009 cases. Currently, VA ESSENCE provides high sensitivity in detecting ILI cases. However, the system's specificity is lower than ideal. Other ESSENCE users, including the U.S. Department of Defense (DoD), have limited their ILI ICD-9-CM codes to enhance the specificity of their system. A study evaluating code-based syndromic surveillance for ILI showed that 14 ICD-9-CM codes (079.99, 382.9, 460, 461.9, 465.8, 465.9, 466.0, 486, 487.0, 487.1, 487.8, 490, 780.6, 786.2) best correlated with positive viral specimens [31]. Four ICD-9-CM codes that are not in the original ILI group (otitis media, acute suppurative otitis media, acute sinusitis, and acute tonsillitis) were also noted to be used during the collection of viral samples [31]. Currently, the DoD ESSENCE system uses viral infection NOS (079.99), otitis media NOS (382.9), acute nasopharyngitis (460), acute sinusitis NOS (461.9), acute upper respiratory infection NOS (465.9), acute bronchitis (466), pneumonia, organism NOS (486), bronchitis (490), fever (780.6), and cough (786.2)[22]. Further chart-review analysis of general symptoms codes including cough and bronchitis would be helpful in distinguishing the degree that chronic diseases contribute to the overall VA ILI rate. Narrowing down the number of ICD-9-CM codes that contribute to the ILI syndrome group may help improve the specificity of VA ESSENCE. The current version also does not include vital signs, lab tests, prescription orders, or markers of severity of illness (i.e. admission or disposition) which could also help to improve the specificity of VA ESSENCE.

There were limitations to our study. Although the mean age of the veteran population is starting to decrease with the return of numerous men and women from Operations Iraqi and Enduring Freedom, at the present time, the veteran population tends to be primarily older males. With pandemic H1N1 2009 influenza, one of the large groups particularly affected are children who are not currently captured in the VA system. The number of confirmed pandemic H1N1 2009 cases in VA has been limited thus far, however, it is likely we can expect to see more cases in the upcoming months despite limited identification of confirmed cases since confirmatory testing is no longer indicated for many patients with suspected pandemic H1N1 2009. Another limitation with the current VA ESSENCE system is the reliance on ICD-9-CM codes. The codes are not assigned until the end of the visit and can take several days to be coded since in general VA provider reimbursement is not directly linked with coding of patient visits. In addition, accuracy of ICD-9-CM codes can be variable, as a previous study evaluating VA's use of ESSENCE to detect hemorrhagic illnesses has illustrated [32]. Therefore the modification of VA ESSENCE to include additional data elements will likely enhance the robustness of the system.

While VA ESSENCE was successful in tracking trends in influenza, several enhancements are planned to help increase specificity while maintaining the innate sensitivity of the system. The addition of other data elements including temperature, laboratory orders for influenza tests, chest x-ray orders, telephone triage data, and pharmacy prescriptions for antivirals and other drugs may help improve sensitivity, specificity, and the ability to predict how severe the potential outbreak may be. Efforts are currently underway to combine VA and DoD ESSENCE biosurveillance data streams which will enhance the population diversity by including a younger active duty military and their dependent population. As described by Lucero *et al.* in a poster presentation at the Association for Professionals in Infection Control and Epidemiology (APIC) conference in 2009, the combining of these two data streams will further improve the system's early detection of outbreaks and influenza trends across all age groups and genders. These future enhancements of VA's ESSENCE will likely improve our current ability to monitor influenza.
